# Effect of organic and inorganic dietary selenium supplementation on gene expression in oviduct tissues and Selenoproteins gene expression in Lohman Brown-classic laying hens

**DOI:** 10.1186/s12917-021-02964-0

**Published:** 2021-08-21

**Authors:** A. I. Muhammad, A. M. Dalia, T. C. Loh, H. Akit, A. A. Samsudin

**Affiliations:** 1 Department of Animal Science, Faculty of Agriculture, Universiti Putra Malaysia, 43400 Serdang, Selangor Malaysia; 2 Department of Animal Science, Faculty of Agriculture, Federal University Dutse, P.M.B. 7156, Dutse, Jigawa State Nigeria; 3 Department of Animal Nutrition, Faculty of Animal Production, University of Khartoum, P.O. Box 321, Khartoum, Sudan

**Keywords:** Organic, Selenium, Gene expression, Oviduct, Selenoprotein, Antioxidant capacity, Laying hens

## Abstract

**Background:**

The oviduct of a hen provides a conducive environment for egg formation, which needs a large amount of mineral elements from the blood via trans-epithelial permeability. Eggshell is the calcified layer on the outside of an egg that provides protection and is critical for egg quality. However, little is known about the genes or proteins involved in eggshell formation, and their relationship to dietary microminerals. We hypothesized that dietary selenium supplementation in chickens will influence genes involved in eggshell biomineralization, and improve laying hen antioxidant capacity. The objective of this research was to investigate how organic and inorganic dietary selenium supplementation affected mRNA expression of shell gland genes involved in eggshell biomineralization, and selenoproteins gene expression in Lohman Brown-Classic laying hens.

**Results:**

Shell gland **(**Uterus) and liver tissue samples were collected from hens during the active growth phase of calcification (15–20 h post-ovulation) for RT-PCR analysis. In the oviduct (shell gland and magnum) and liver of laying hens, the relative expression of functional eggshell and hepatic selenoproteins genes was investigated. Results of qPCR confirmed the higher (*p* < 0.05) mRNA expression of OC-17 and OC-116 in shell gland of organic Se hen compared to inorganic and basal diet treatments. Similarly, dietary Se treatments affected the mRNA expression of OCX-32 and OCX-36 in the shell gland of laying hens. In the magnum, mRNA expression of OC-17 was significantly (*p* < 0.05) higher in hens fed-bacterial organic, while OC-116 mRNA expression was down-regulated in dietary Se supplemented groups compared to non-Se supplemented hens. Moreover, when compared to sodium selenite, only ADS18 bacterial Se showed significantly (*p* < 0.05) higher mRNA levels in GPX1, GPX4, DIO1, DIO2 and SELW1, while Se-yeast showed significantly (*p* < 0.05) higher mRNA levels in TXNRD1 than the non-Se group.

**Conclusions:**

Dietary Se supplementation especially that from a bacterial organic source, improved shell gland and hepatic selenoproteins gene expression in laying hens, indicating that it could be used as a viable alternative source of Se in laying hens. The findings could suggest that organic Se upregulation of shell gland genes and hepatic selenoproteins in laying hens is efficient.

## Background

Selenium (Se) is a trace element that is necessary for a variety of physiological functions in animals, including immunoregulatory function [1, 2], reproduction [3], and protecting the tissue damage by the maintenance of the antioxidant system [4, 5]. Se-containing proteins are primarily responsible for the mechanisms by which Se functions [6]. Selenocysteine is the major part by which Se exerts its biological role within a living system after incorporation into selenoproteins [7]. Consequently, selenoprotein levels and selenoprotein mRNA yield are affected by Se supply. More than 25 unique selenoproteins have been identified in chickens, all of which play key roles in the catalytic activity site. Glutathione peroxidases, iodothyronine deiodinases, and thioredoxin reductase are some of the enzymes that have been discovered in humans and animals [8].

Nutritional form and levels of Se supplementation in the diet influence selenoproteins synthesis [9, 10]. A substantial number of studies have found a link between dietary Se supplementation and selenoproteins expression in animal tissues. The GPX activity signals as a biomarker of Se status [11], and a Se-deficient diet suppresses selenoproteins expression in the broiler muscular stomach [12]. Similarly, Zhang et al. [13] found that the low-Se diet group had downregulated mRNA levels of 14 selenoproteins genes and upregulated mRNA levels of 9 selenoproteins genes, but no effect on DIO3 nor SELENOPX1 mRNA levels in broiler kidney. In chicken liver, excess Se was found to down-regulate the expression of GPX4 mRNA [14]. Furthermore, broiler fed a sodium selenite-supplemented diet for 90 days showed an elevation in SELENOW1 liver mRNA [15].

Recent advances in genomic technology, mainly using species-specific microarrays, have the potential to help investigation and clarify how nutrients influence gene expression profiles, thus influencing cellular functions. It could be used to provide valuable data on how different forms of nutrients modulate their consequences on production and reproduction. The oviduct of laying hen is considered a biologically conducive environment for egg development and potential fertilization [16]. The biological process of mineralization of the hen eggshell is extremely complex, but it has resulted in efficient calcium mobilization and biomineralization [16], from the bloodstream via the uterine trans-epithelial cells, and finally into the uterine fluid, which bathes the eggshell [17]. Furthermore, gene activation in the biological process of calcification is tissue-specific and time-dependent [17, 18]. The egg’s formation occur in the chicken oviduct and is highly complex and genetically and hormonally regulated, with many genes and biological pathways involved [16, 19]. The magnum is the oviduct’s largest segment, producing the egg-white proteins that encapsulate the yolk [20]. Its glandular epithelial cells synthesize various egg-white proteins that are stored and released only for the 2–3 h that the egg remains. The egg descends into the shell gland (uterus) and remains there for averagely 18–22 h during which the calcite crystals are deposited for complete mineralization of the eggshell. The functions of many genes and proteins in eggshell synthesis and mineralization have been extensively studied. The eggshell mineralization is activated with the formation of calcite nodules and occurs in an acidic medium in the extracellular matrix uterine fluid [20]. Ovocleidins (OC), ovocalyxins (OCX), and osteopontin (OPN) are matrix proteins that play a key role in the organization of the calcite crystals during eggshell calcification [16, 17, 21]. OC’s are eggshell matrix proteins that regulate the uterine crystallization process. OC-17 which catalyzes the mineralization of amorphous calcium carbonate to calcite crystals, while OC-116 regulates the organization of calcite crystals in the eggshell [22]. Similarly, OCX’s are a family of three proteins that play a role in eggshell mineralization. OCX-32 regulates the morphology of calcite crystals and functions as an anti-mineralizer during the calcification termination process [23], while OCX-36’s direct function in eggshell calcification is to protects the egg from microbial invasion [24]. Also, ovocalyxins, OCX-21, is another member that ensures high-quality eggshell formation [25].

The Se form and the length of time it is supplemented has a major effect on poultry reproductive performance [26, 27]. Se supplementation (< 8 weeks) of any form or source has been found to have effect on hen reproductive parameters [28, 29]. In broiler breeder hens’ [30, 31], layers [32, 33], and duck breeders [34], birds supplemented with organic Se for more than 12 weeks increased egg production, fertility, hatching efficiency. Nevertheless, it is clear that Se supplementation affects hen reproductive performance, little is known about how it modulates these effects. According to previous research, the sources of Se and their levels in animal tissue may have a wide range of metabolic effects [17, 35]. Besides, the Se bioavailability of either source and form is determined by the absorption pathways [36]. Different strains of microorganisms may be used to produced organic Se through the microbial reduction pathway. *S. maltophilia* (ADS18), for example, was isolated from hot-spring water and found to have a high concentration of organic Se-containing proteins, making it ideal for use as a Se source in poultry [37]. Although, it is evident that Se can enhance the antioxidant system, scientific data on the effect of this new organic Se source on layers is scarce, and to our knowledge, no published study has reported to investigate the effect of bacterial organic Se from ADS18 source on the expression of shell gland genes and selenoproteins in layers. Therefore, the present study was designed to investigate how organic and inorganic dietary selenium supplementation affected mRNA expression of shell gland genes involved in eggshell biomineralization, and selenoproteins gene expression in Lohman Brown-Classic laying hens.

## Results

### Effects of dietary selenium supplementation on mRNA expression of eggshell matrix and other proteins in shell gland

**Fig. 1:**
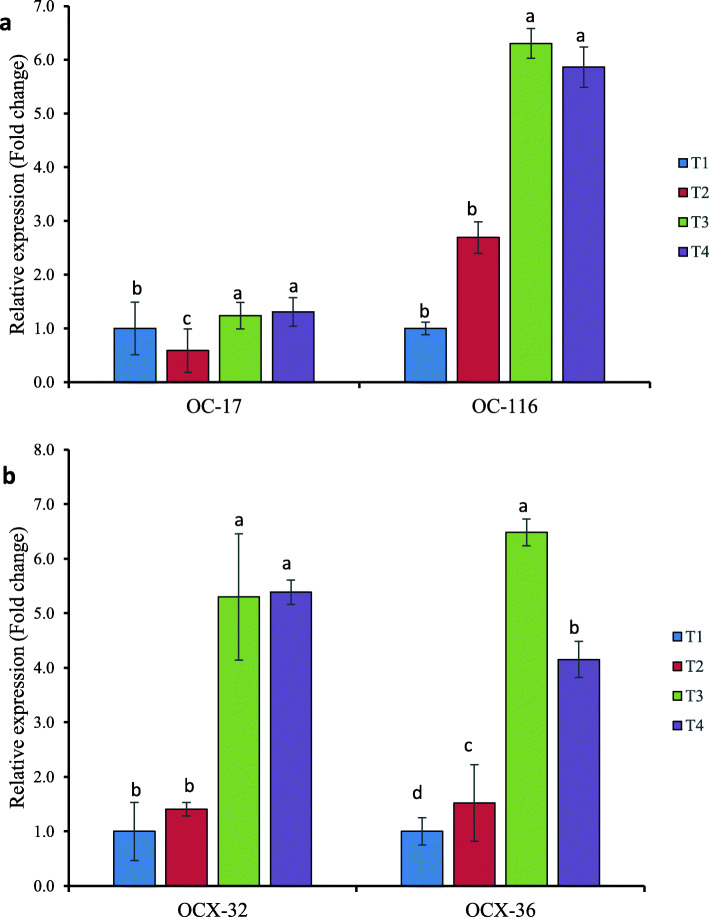
Relative expression levels of the calculated target genes affected by different dietary Se source in the shell gland of laying hens. **a** OC-17 and OC-116 mRNA expression. **b** OCX-32 and OCX-36 mRNA expression. The fold changes were normalized with a housekeeping gene (GADPH and β-actin). Then, treated samples were expressed relative to the gene expression of the CON group (T1). Data represented as the means ± standard error. Treatments: T1; basal diet, T2 basal diet + 0.3 mg/kg sodium selenite, T3: basal diet + 0.3 mg/kg Se-Yeast, T4: basal diet + 0.3 mg/kg Se of ADS18. Primer pairs used for these analyses are listed in Table 2

OC-17 and OC-116 mRNA expression in the shell gland differed significantly (*p* < 0.05) between the experimental groups (Fig. 1a). Organic Se (T4 or T3) supplemented hens had higher (*p* < 0.05) mRNA expression of OC-17 and OC-116 than inorganic Se (T2) and the negative control (T1). In comparison to hens fed organic Se (T4 or T3) or a negative control (T1), the expression of OC-17 mRNA was down-regulated in inorganic Se (T2) fed hens. However, in organic Se (T4 or T3) supplemented hens shell gland, mRNA expression of OC-116 was higher (*p* < 0.05) than in sodium selenite (T2) or non-supplemented (T1) groups. Furthermore, dietary Se treatments impacted the laying hens ‘shell gland mRNA expression of OCX-32 and OCX-36(Fig. 1b). Only the organic Se treated group (T4 or T3) demonstrated significantly higher (*p* < 0.05) OCX-32 mRNA expressionthan the inorganic (T2) and non-Se supplemented (T1) groups. The Se-yeast (T3) supplemented group had the greatest levels of OCX-36 mRNA expression, followed by bacterial selenoprotein (T4), and sodium selenite (T2) fed hens, and the lowest levels in non-Se supplemented (T1) laying hens shell gland.

### Effects of dietary selenium supplementation on mRNA expression of eggshell matrix and other proteins in magnum

**Fig. 2:**
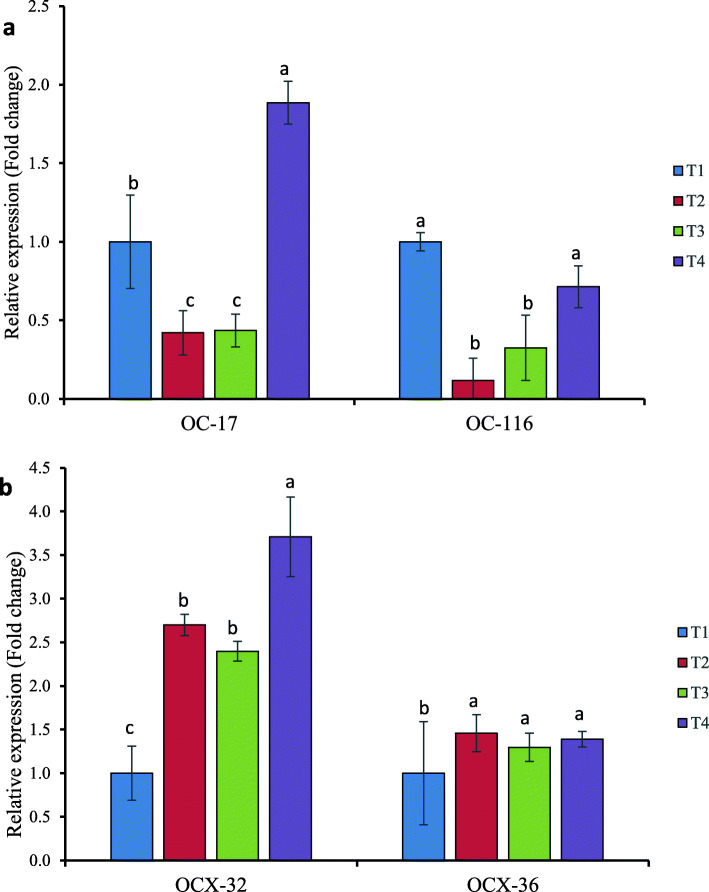
Relative expression levels of the calculated target genes affected by different dietary Se source in the magnum of laying hens. **a** OC-17 and OC-116 mRNA expression (**b**) OCX-32 and OCX-36 mRNA expression. The fold changes were normalized with a housekeeping gene (GADPH and β-actin). Then, treated samples were expressed relative to the gene expression of the CON group (T1). Data represented as the means ± standard error. Treatments: T1; basal diet, T2 basal diet + 0.3 mg/kg sodium selenite, T3: basal diet + 0.3 mg/kg Se-Yeast, T4: basal diet + 0.3 mg/kg Se of ADS18. Primer pairs used for these analyses are listed in Table 2

Figure 2a, b shows the effect of dietary Se supplementation on eggshell proteins gene expression in the magnum of laying hens as measured by real-time PCR. Only in hens supplemented with bacterial organic Se (T4) did the expression of OC-17 mRNA up-regulated (*p* < 0.05) (Fig.2a). Hens fed Se-yeast (T3) and sodium selenite (T2), on the other hand, showed a down-regulation of the same gene (OC-17), with no significant (*p* > 0.05) difference between two groups, and were lower than the non-supplemented (T1) group. In contrast to the non-Se supplemented group, all of the dietary Se treated groups had lower mRNA expression of OC-116. Despite the fact that bacterial selenoprotein group (T4) downregulates OC-116 mRNA, it is not statistically different (p > 0.05) from negative control (T1), and both (T1 and T4) are statistically different (*p* < 0.05) from (T3) and (T2), respectively. Furthermore, the quantitative expression of OCX-32 and OCX-36 in magnum demonstrated that dietary Se treatments influenced mRNA levels (Fig. 2b). OCX-32 mRNA expression was up-regulated in all dietary Se treatments, with the highest expression in bacterial organic Se (T4). OCX-32 mRNA expression did not differ significantly (*p* > 0.05) between Se-yeast (T3) and sodium selenite (T2) fed hens, however, it was significantly better (*p* < 0.05) in the magnum of negative control (T1) hens. The dietary Se treated groups (T2 – T4) had no significant difference (p > 0.05) in OCX-36 mRNA expression, however non-Se supplemented (T1) hens had lower (*p* < 0.05) OCX-36 mRNA expression in their magnum.

### Effects of dietary selenium supplementation on mRNA expression of hepatic selenoproteins in the liver of laying hens

**Fig. 3:**
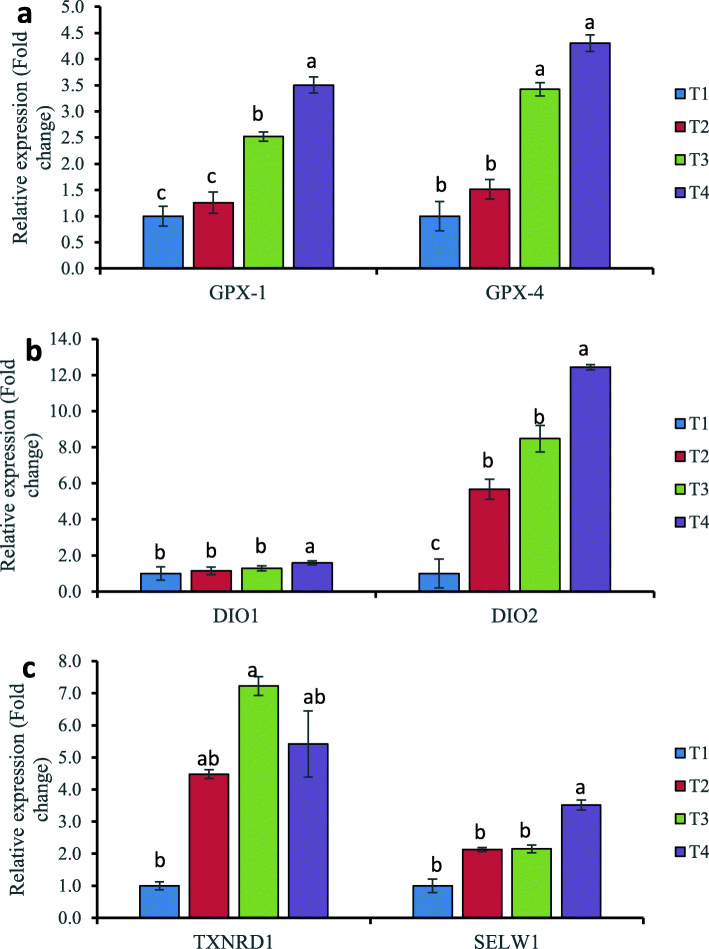
Relative expression of selenoprotein transcripts liver of laying hens fed different dietary Se source. **a** GPX1 and GPX4 mRNA expression. **b** DIO1 and DIO2 mRNA expression. **c** TXNRD1 and SELENOW1 mRNA expression. The fold changes were normalized with a housekeeping gene (GADPH and β-actin). Then, treated samples were expressed relative to the gene expression of the CON group (T1). Data represented as the means ± standard error. Treatments: T1; basal diet, T2 basal diet + 0.3 mg/kg sodium selenite, T3: basal diet + 0.3 mg/kg Se-Yeast, T4: basal diet + 0.3 mg/kg Se of ADS18. Primer pairs used for these analyses are listed in Table 2

The relative expression of some selenoproteins of hens supplemented with organic and inorganic dietary Se sources is shown in Fig. 3a, b. The hepatic expressions of GPX1, GPX4, DIO1, DIO2, TXNRD1, and SELW1 genes were investigated. Dietary Se supplementation altered the expression of GPX1 and GPX4 (Fig. 3a). Organic Se (T4 or T3) supplemented hens had significantly (*p* < 0.05) higher expression of both GPX1 and GPX4 than the inorganic and non-supplemented groups. GPX1 levels were significantly (*p* < 0.05) higher in T4 than T3, and other treatment groups. Similarly, organic (ADS18 or Se-Yeast) Se showed higher (*p* < 0.05) fold changes in mRNA expression of GPX4 than inorganic (sodium selenite) and control groups. GPX1 and GPX4 mRNA expression were higher in hens fed an inorganic Se-supplemented diet (T2) than in the negative control, albeit the differences were not statistically significant (*p* > 0.05).

The fold changes in the relative expression of DIO1 and DIO2 mRNA levels in the liver tissue are shown in Fig. 3b. T4 (ADS18) had a higher fold change in relative gene expression of both genes,with significant differences between treatment groups. Only bacterial selenoprotein (T4) was found to be significantly (*p* < 0.05) different from other treatment groups; however, DIO1 mRNA expression in T3 and T2 was marginally higher than the negative control but not significantly (p > 0.05) different. A significant (*p* < 0.05) increase in DIO2 mRNA expression was observed in the liver of hens supplemented with bacterial organic Se compared to hens fed Se-yeast (T3), sodium selenite (T2), and negative control (T1). Regardless of Se forms, Se supplemented hens demonstrated higher fold changes in DIO1 and DIO2 hepatic mRNA expression.

The mRNA expression of TXNRD1 and SELW1 genes in livers of hens fed different forms of dietary Se was shown in Fig. 3c. TXNRD1 mRNA fold changes were significantly (*p* < 0.05) different in the T3 (Se-yeast) treatment group’s liver, which differed slightly from the negative control only. In either of the dietary Se supplemented groups, however, there was no significant (p > 0.05) difference in liver mRNA levels. Furthermore, all of the Se supplemented groups had a significant fold change in hepatic SELENOW1 mRNA level, with no effect when compared to the basal diet with no Se supplementation. Despite the fact that the Se supplemented group (T2, T3, and T4) had a higher fold change than the negative control group (T1), hens fed bacterial organic Se (T4) showed a significant (*p* < 0.05) difference from the other treatment groups.

## Discussion

One of the most critical roles of the eggshell is to enfold and shelter the egg contents through its mechanical properties for optimum economic success in layer production [44]. The ultrastructure of hen eggshells is a highly ordered structure with unique mechanical properties, crystal morphology and organic matrix [45, 46]. Calcium carbonate has 95% calcitic polymorph and 3.5% organic matrix macromolecules. Physiological changes and the complex stages of egg calcification, which include uterine cells and fluid constituents, are among the many factors that affects its formation [17, 45]. The formation of complex bio-ceramic eggshell arises from direct acellular uterine fluid interaction of ions such as Ca^2+^ and HCO_3_^−^ and precursors of organic constituents [23, 45], with an uninterrupted action of cells [47]. Soluble precursors like proteins and minerals were released by shell gland cells into the acellular uterine fluid [46]. A solid layer is formed as a result of interaction between the developing crystal and organic shell matrix with highly systematic microstructure and texture as eggshell by extraordinary mechanical properties [17, 45]. The current study compared gene expression in the oviduct of laying hens supplemented with various Se sources. Egg formation and yolk ovulation are stimulated by reproductive hormones during the active calcification stage, thereby, regulating the calcium metabolism [46]. Furthermore, genes involved in the biomineralization process and/or supply of shell precursors may be upregulated. The chicken shell gland plays a key role in the daily calcification of the shell during 19 h process though the egg remains in there. Moreover, a compact layer is formed due to the interaction between the developing crystal and organic shell matrix with largely systematic microstructure and texture as eggshell by great mechanical properties [23, 45]. This research focused primarily on genes/proteins that may be involved in the biomineralization process. Interactions between these proteins and crystal formation have been demonstrated in numerous studies [17]. However, studies on the effects of different Se sources on the expression of reproductive genes were not thoroughly researched, and there was no published data on the efficacy of bacterial organic Se on laying hens to our knowledge. In the current study, dietary Se supplementation affects the mRNA expression of all the examined genes by either up-regulation or down-regulation depending on the type of tissue. Physical egg quality factors such as shell thickness, egg shape, and elasticity are determined by the mRNA expression of OC-116 jointly with OCX-32 genes rather by these protein abundance [45, 48]. The absence of these matrix proteins can result in the complete cessation of the mineralization process [19]. Fragile, shape stiffness, and thickness of eggshells are linked to irregular OC-116 gene expression or OC-116SNP variants [49].

The chicken ovocleidin gene (OC-17 and OC-116) is expressed in the shell gland as one of the potential eggshell matrix proteins [16, 19]. They were both characterized as soluble and insoluble eggshell matrix proteins. Both were identified as framework proteins that aligned calcite crystals during the mineralization process [45]. The OC-17 protein has an antimicrobial function and regulates the biomineralization process [50, 51]. Ovocleidin-116 is a major component of the chicken eggshell matrix extracellular phosphoglycoprotein, which is abundant in uterine fluid during the active phase of calcification, and is therefore thought to function in the mineralization of the eggshell [52–54]. OC-116 is involved in eggshell and bone strength mineralization [52, 55, 56], and regulates the arrangement of calcite crystals in eggshell [16]. Regardless of the treatment groups, the results showed higher mRNA expression of OC-17 and OC-116 in the shell tissue than in magnum. This was confirmed by published data [19, 44, 46, 57], who found higher OC-116 genes mRNA expression in the shell gland tissue. Furthermore, there has been an interaction between organic matrix and inorganic minerals in chicken uterine fluid, resulting in tough and calcification of eggshell [19].

The shell of an egg is considered a physically protective wall for the contents from external (microbial) inversion and is rich in proteins with antimicrobial properties [46]. These antimicrobial properties are contained in the liquid egg-white, and potentially present chemical defensive mechanisms to the egg [58]. The lumen is sheltered from bacteria-free, thus protect the forming egg or embryo by antimicrobial proteins released into the uterine fluid. Ovocalyxin-32 and 36 are two antimicrobial proteins expressed in the tissues during the shell calcification that have been studied. Ovocalyxin family (OCX-32 and OCX-36) constitutes mostly organic matrix proteins [17, 46], highly expressed by uterine glandular cells, eggshell membrane and, egg vitelline membrane especially during the active calcification phase [39]. In avian *spp,* ovocalyxin-32 is the main determinant of eggshell quality, while ovoaclyxin-36 presents an antimicrobial property integrated into the eggshell [24]. Also, antimicrobial properties were discovered in the recombinant of OCX-32 [59]. Besides, OCX-36 belongs to the lipopolysaccharide-binding proteins and Bactericidal Permeability Increasing (BPI) family, and it is recognized to play a role in anti-bacterial defense in mammals [46]. Members of these family might be lethal to Gram-negative bacteria via binding to the lipid A portion of the lipopolysaccharide cell wall. The current study found that regardless of dietary Se treatments, mRNA expression of OCX-32 and OCX-36 was up-regulated in the shell gland than in magnum tissue. Poyatos et al. [57] and Yin et al. [44] identified highly expressed OCX-32 precursors in the shell gland with egg, which is consistent with our findings. Similarly, Yin et al. [60] found that the OCX-32 gene was expressed higher in the distal oviduct (isthmus and shell gland) than in the proximal oviduct (magnum and shell gland), suggesting that it is secreted by the glandular epithelium of the shell gland [61]. Similarly, Jonchère et al. [61] and Brionne et al. [46] established that OCX-36 is shell gland specific, and increases through the calcification of eggshell. On the other hand, OCX-32 was highly expressed in isthmus than ovary and magnum, although lower than the uterus [44]. Because of its similarity to lipopolysaccharide-binding proteins and bactericidal permeability-increasing protein, OCX-36 expression has been discovered in the isthmus and shell gland, and is thought to participate in natural defense mechanisms [44]. The findings of Hrabia et al. [62] suggest that growth hormones may participate in the expression of some oviduct specific proteins (OCX-32 and OCX-36) in the chicken. Brennan et al. [27] discovered that different Se forms supplementation changes the expression patterns of genes involved in energy production and protein synthesis pathways in the oviduct gene expression profiles of broiler-breeder hens. However, the gene expression of OCX32 and OC116 in the shell gland of 85-week-old laying hens reduced compared to 75-week-old laying hens fed varied dietary vitamin supplementation levels [63]. Microminerals such as cadmium (Cd) supplementation were found to reduce the expression of ovocalyxin-32 (OCX-32), ovocalyxin-36 (OCX-36), osteopontin (SPP1), and ovocledidin-17 (OC-17), which has a deleterious impact on egg quality and eggshell deposition in laying hens by disrupting the metabolism of eggshell glands [64]. Nonetheless, little is known about the mechanisms through which Se exerts its physiological effects in reproductive tissue, necessitating additional research to uncover the fundamental mechanisms that underpin these responses.

The present study investigated the expression of selenoproteins in the liver of laying hens fed with two forms of organic Se from bacteria and yeast compared with an inorganic source (sodium selenite). The liver is the primary organ and site for nutrients (carbohydrate, protein, and fat metabolism) homeostasis [65]. The up-regulation and down-regulation of selenoproteins mRNA expression is dietary Se ingestion dependent [66]. Selenium’s physiological and biological functions have been stated to be mediated primarily via the activity of selenoproteins [15, 67], and its deficiency has been linked to chemical and biological dysfunction [67]. In the current research, organic Se significantly upregulated the mRNA levels of hepatic selenoproteins in hen’s liver as compared to inorganic or non-supplemented groups, implying that organic Se could have antioxidant properties and thus reduced oxidative stress [68]. Contrary to inorganic Se, it is passively absorbed into the system with typical lower absorption rates [69]. Those results are consistent with previous studies. The foremost selenoproteins discovered and abundant in the liver are GPX (GPX1–4), has enzymatic properties, with the majority of them involved in peroxides catabolism [70, 71] Hou et al. [72] reported Se-enriched *S. cerevisiae* (SSC) supplementation significantly increased GPX1 and GPX4 expression levels in broiler chicken muscle compared with control, *S. cerevisiae,* and sodium selenite group. Recently, Chen et al. [73] reported selenide chitosan sulfate (Se-CTS-S) up-regulate GPX1 and GPX4 mRNA levels in hepatocytes and liver of chickens compared with chitosan (CTS), chitosan sulfate (CTS-S), selenide chitosan (CTS-Se), and sodium selenite (Na_2_SeO_3_). Meng et al. [4] found that laying hens supplemented with nano-Se and Se-yeast had higher GPX1 and GPX4 mRNA levels in their livers, respectively. Wang et al. [74] reported dietary Se-yeast supplementation caused an up-regulation of selenoproteins gene expressions in the liver (10) and muscles (11) of rainbow trout (*O. mykiss*). Similarly, organic bacterial Se showed a significant increase in liver mRNA expression of GPX1, GP4, DIO1, and TXNRD1 compared to sodium selenite supplemented broilers [10]. Chen et al. [75] reported higher expression of GPX1 and GPX4 mRNA levels with organic Se supplementation of Se-enriched *S. cerevisiae* compared to other groups in *Arbor Acres* broilers. Khan et al. [76] observed the upregulation of mRNA expression of GPX1 and GPX4 and downregulation of heat shock proteins genes (HSP60, HSP70, and HSP90) in the chicken heart with Se-enriched probiotics. Luan et al. (2016) found lower selenoproteins transcript levels in chicken erythrocytes fed a Se-deficient diet, but high expression of GPX, TXNRD1, selenoprotein P1 (SELENOP), and selenoprotein synthetase (SPS2) compared to other selenoproteins. The GPX family (GPX1, GPX2, GPX3, and GPX4) is abundant in the liver, where it catabolizes peroxides. For instance, GPX1 is a potential antioxidant enzyme with a significant role in the detoxification of lipid hydroperoxides and H_2_O_2_, while GPX4 reduces oxidative stress and thus, inhibits atherosclerosis [76]_._ Previous research has shown that dietary Se intake increased GPX and SELENOW1 mRNA levels in poultry [67], and sheep [77]. However, there were no results on the efficacy of bacterial organic Se on laying hens, and no research on the effect of different Se sources on the expression of these genes. The bioavailability of Se sources or forms and levels differs by tissue type and animal species in terms of absorption, deposition, and metabolism, which could affect antioxidant enzyme activities directly or indirectly [78]. As a consequence, ADS18 or Se-yeast supplementation may be related to the regulation of GPX’s, resulting in reduced body oxidative stress through the transcription level of GPX1 and GPX4 mRNA in the liver of laying hens. It is possible that the results indicate that GPX1 is more responsive to Se regulation than GPX4, and that the responses to dietary Se mRNA expression vary between the two selenoproteins. Moreover, it can protect from Se-deficiency disorder [79]. Furthermore, mRNA expression of GPX1 and GPX4 can be used as molecular biomarkers for evaluating Se status and the requirements [80].

The Iodothyronine deiodinase (DIO) family plays an essential role for thyroid metabolism [81], and thioredoxin reductase (TXNRD) genes which constitute a major cellular redox system in all living organism [82]. Furthermore, the qPCR analysis showed that the relative higher mRNA levels of DIO1, DIO2, TXNRD1, and SELW1 genes were expressed in the liver, an organ that is more responsive to changes in dietary Se levels and forms [83]. The results indicated that Se sources and intake alter the mRNA levels of laying hens selenoproteins, and the effects vary greatly between different selenoproteins and tissues [83, 84], although only liver tissue was investigated in this study. Lin et al. [84] reported downregulation of DIO1, DIO2, DIO3, TXNRD2 selenoproteins induced by Se deficiency in chicken’s thyroid gland. Also, Liu et al. [85] observed downregulation of the SEPW1 mRNA level in pig’s liver fed a high-Se diet of 3.0 mg Se/kg against the 0.3 mg Se/kg diet. Similarly, hepatic expression of GPX1, SELENOPW1, and SELENOPW15 mRNA levels were decreased by dietary Se deficiency in chicks liver and muscle [80], SelW in layers liver [15]. Conversely, supplementation with the inorganic form of Se (sodium selenite) causes higher levels of mRNA expression of GPX1, SELENOW1, SELENOP15, and TXNRD1 in lamb liver, while GPX4 is unaffected [83]. The transcripts DIO1, DIO2, TXNRD1, and SELENOW1 were all found to be upregulated in the livers of Se supplemented hens. In agreement with these findings, TXNRD and GPX were found to be effective in reducing free radical-mediated peroxidation and redevelopment in male Wistar rats following Se supplementation [86]. Accordingly, higher Se supplementation may be responsible for preserving optimal activities of GPX and TXNRD, and partial detoxification against the negative effects of Cd in male rats [87, 88], and broilers [89]. A recent trial on the toxicity of Pb revealed that Se might alleviate the downregulation of GPX4, 2 and 1, DIO1, DIO2, TXNRD2–3, selenoprotein U, I, O, M, K, W, T, S 15 SEPX1, and SEPP1 expression in chicken cartilage tissue [90, 91]. Similar results with Se-yeast and SeMet as organic Se sources upregulate GPX1 and TXNRD1 mRNA expression in broiler breeders compared with sodium selenite [35, 92]. Furthermore, SELW1 may participate in the protective role against H_2_O_2_, oxidative stress, and metabolic pathways [83, 91, 93], as comparable data were published in rat testes [94] and pig liver [67]. It is noteworthy, that the findings showed a clear trend of up-regulating selenoproteins (GPX1, GPX4, DIO1, DIO2, and SELENOW1) mRNA expression significantly with bacterial organic Se supplementation, except for TXNRD1 with Se-yeast hens compared to the negative control. Furthermore, the findings suggested that DIO2 mRNA may be more sensitive to regulation to bacterial organic Se status than others, and that mRNA expression responses to dietary Se source may vary between selenoproteins (GPX1, GPX4, DIO1, DIO2, TXNRD1, and SELENOW1). The noted variance between organic and inorganic Se may be explained by organic forms higher bioavailability, which stimulates more selenoproteins gene expression [95]. Surai et al. [96] and Meng et al. [4] also suggested the mechanisms of action behind nano-Se are by the mediation of the gut microbiota in converting nano-Se into selenite, H_2_Se, or Se-phosphate with the synthesis of selenoproteins. Organic Se compounds such as; SeMet, SeCys, and Se-methyl-Se cysteine among others, have been shown to have different bioavailability in the body [97]. Moreover, the current study notes a significant change in mRNA expression of liver selenoproteins in the hens regardless of Se source or form. However, the mechanisms behind how different Se sources regulate selenoproteins expression remain unknown and require further exploration.

## Conclusion

In conclusion, the current study showed that the expression of uterine genes and selenoproteins was upregulated by basal diets supplemented with 0.3 mg/kg of different organic sources of Se and sodium selenite. Compared to inorganic and non-Se supplemented hens, the bacterial selenoprotein proves stronger at increasing the expression of functional genes involved in the formation of eggs (eggshell biomineralization) and selenoproteins.

## Methods

### Animal ethics and husbandry management

All procedures involving animal care, handling, and sampling were performed in compliance with the guidelines and regulations and were approved by the Universiti Putra Malaysia’s Institutional Animal Care and Use Committee (UPM/IACUC/AUP-R063/2018) before the commencement of the research. A total of one 144 Lohman Brown-Classic laying hens (initial live weight 1714 ± 185 g) of 23 weeks-old were randomly divided into four homogenous groups of 36 hens each, as well as six replications of six hens each. The hens were raised in a two-tier stainless-steel cage with a bird per cage in an open ventilated henhouse at Universiti Putra Malaysia’s Ladang 15 Poultry Unit in Serdang. The cage measured 30 cm *×* 50 cm *×* 40 cm (width depth height).

### Experimental diets

A corn and soya bean-meal as a basal diet for laying hens was prepared (Table 1) according to NRC (National Research Council) [98] guidelines, except for Se which was supplemented as 0.3 mg/kg feed according to Surai [99]. Three supplemented diets were tagged as basal diet plus 0.3 mg/kg feed inorganic sodium selenite (Na_2_SeO_3_), basal diet plus 0.3 mg/kg selenium yeast (Se-Yeast), and basal diet plus 0.3 mg/kg ADS18 enriched bacterial protein. The preparation of the bacterial Se is described by Dalia [100]. The experimental diets were prepared monthly and kept in capped plastic containers at room temperature. The hens were limited to 120 g/hen/day to reduce the feed-selection behaviour seen in laying hens. During the experimental period, the feed was offered once a day (07:00–08:00) with ad libitum access to water and treatment diets at an ambient temperature of about 30 ± 5 °C. A lightening schedule of 16-h light and 8-h dark was practiced, with light beginning at 17:00 local time following the Lohman management guide [101]. The experiment lasted for sixteen (112 days) weeks excluding 4 weeks of adaptation.

**Table 1:** Ingredient composition and analyzed nutrient levels of the basal diet (on dry matter basis)

Ingredients	Con	Na_**2**_SeO_**3**_	Se-Yeast	ADS18
Corn	44.00	44.00	44.00	44.00
Soybean meal 48%	29.00	29.00	29.00	29.00
Wheat pollard	11.00	11.00	11.00	11.00
CPO	3.50	3.50	3.50	3.50
L-Lysine	0.10	0.10	0.10	0.10
DL-methionine	0.25	0.25	0.25	0.25
Dicalcium phosphate (18%)	2.00	2.00	2.00	2.00
Calcium carbonate	7.70	7.70	7.70	7.70
Choline chloride	0.10	0.10	0.10	0.10
Salt	0.35	0.35	0.35	0.35
Mineral mix^a^	0.60	0.597	0.597	0.597
Vitamin mix^b^	0.60	0.60	0.60	0.60
Antioxidant^c^	0.40	0.40	0.40	0.40
Toxin binder^d^	0.40	0.40	0.40	0.40
Sodium selenite	0.00	0.003	0.00	0.00
Se-yeast	0.00	0.00	0.003	0.00
ADS18-Bacteria	0.00	0.00	0.00	0.003
**Total**	**100**	**100**	**100**	**100**
**Analyzed composition**				
Metabolizable energy Kcal/Kg	2761.24	2761.24	2761.24	2761.24
Crude protein (%)	17.66	17.66	17.66	17.66
Crude fat (%)	5.3	5.3	5.3	5.3
Fibre (%)	3.98	3.98	3.98	3.98
Calcium (%)	3.65	3.65	3.65	3.65
Total Phosphorus (%)	0.88	0.88	0.88	0.88
Av. Phosphorus for poultry (%)	0.48	0.48	0.48	0.48
Analysed Se (mg/kg)^e^	0.03 ± 0.01	0.31 ± 0.02	0.32 ± 0.01	0.33 ± 0.02

^a^Mineral premix provided (per kg of diet): Iron 120 mg, Manganese 150 mg, Copper 15 mg, Zinc 120 mg, Iodine 1.5 mg, and Cobalt 0.4 mg.^b^Vitamin premix supplied (per kg of diet): Vitamin A (retinyl acetate) 10.32 mg, Cholecalciferol 0.250 mg, Vitamin E (DL-tocopherol acetate) 90 mg, Vitamin K 6 mg, Cobalamin 0.07 mg, Thiamine 7 mg, Riboflavin 22 mg, Folic acid 3 mg, Biotin 0.04 mg, Pantothenic acid 35 mg, Niacin 120 mg and Pyridoxine 12 mg. ^c^Antioxidant contains butylated hydroxyanisole (BHA). ^d^Toxin binder contains natural hydrated sodium calcium aluminum silicates to reduce the exposure of feed to mycotoxins. Feed live International Software (Nonthaburi, Thailand) was used to formulate the diets. ^e^The Se content measured using ICP.MS.

### Slaughtering and tissues sampling

A total of twenty-four hens were selected randomly (hen per each replicate) and slaughtered according to the Halal procedure, as described in the Malaysian Standard [102]. Before slaughter, hen’s abdominal palpation was used to assume the egg presence in the shell gland. The carcass was skinned ventrally, and uteri and magnum samples were collected from hens at the active growth phase of calcification (15–20 h post-ovulation) for RT-PCR. It is aimed at targeting higher expression of genes responsible for eggshell biomineralization an egg. Sections of the uterine tissues were scrapped for total RNA isolation, transferred into 5 ml capped tubes, and immediately snapped frozen in liquid nitrogen and stored at −80 °C before extraction of RNA. Furthermore, a portion of liver tissue was sliced and frozen directly in liquid nitrogen and stored at − 80 °C to await analysis.

### Total RNA isolation and purification

Total RNA was isolated from frozen tissues (shell gland, magnum and liver) (30 mg) using RNeasy® Mini Kit (Cat. No. 74104, Qiagen, Hilden, Germany) according to manufacturer’s instructions. The purity and concentration of total RNA was determined using Thermo Multiskan® GO (Thermo, USA) and only samples with an RNA quality > 1.9 were further used for quantitative real-time PCR.

### Quantitative real-time RT-PCR (qPCR) for uterine and selenoprotein mRNA expression

The real-time polymerase chain reaction was performed with the Bio-Rad CFX Manager™ 3.1 real-time PCR system (Bio-Rad Laboratories, Hercules, CA, USA), in 96-well optical reaction plates. Primers used were designed (*HuaGene*™, MyTacg Bioscience Malaysia) according to published *G. gallus* sequences Table 2. The synthesis of first strand cDNA was run by reverse transcription of 1 μg isolated total RNA (20 μl reaction mixture) using QuantiNova Rev. Transcription Kit (cat. No. 205413, Qiagen, Hilden, Germany). The reaction was done in a Bio-Rad thermal cycler (MyCycler, Germany). Master mix was prepared as per the manufacturer’s protocols. Real-time PCR was then performed using QuantiNova SYBR Green PCR Kit (cat. No. 208054, Qiagen, Hilden, Germany) on a Bio-Rad CFX Manager™ 3.1 real-time PCR system (Bio-Rad Laboratories, Hercules, CA, USA). Each reaction (20 μL) contained 10 μL QuantiNova SYBR Green Master Mix, 1 μL of each forward and reverse primers, 7 μL of nuclease-free water and 1 μL of cDNA. The qPCR reactions were carried out following standard cycling mode as per kit protocol. A melting curve was also generated to confirm the sequence-specific PCR products. Three house-keeping genes of Glyceraldehyde 3-phosphate dehydrogenase (GAPDH), Beta-actin (β-actin) and TATA-Box Binding Protein (TBP) were used in triplicates in each tissue of each hen of the experiments to determines the stable house-keeping gene in tissues. The target genes were analyzed in duplicates and their expression level was determined using cycle threshold (Ct) values following standard curve method after normalization with reference genes. Genes of interest were amplified through the following thermo cycling program: reverse transcription at 95 °C for 10 min, first denaturation at 95 °C for 2 min, then 40 cycles of denaturation at 95 °C for 5 s, and combined primer annealing/extension at 60 °C for 10 s. The fluorescent data were acquired at the end of each annealing step during PCR cycles with a construct of melting curve to assess the specificity of PCR amplification. A real-time PCR was run for each pair of primer in which cDNA samples were replaced with distilled water to ascertain the absence of exogenous DNA. The efficiency of amplification was determined for each primer pair using cDNA serial dilutions utilization. The fold changes for each target gene was calculated using power of 2 ^(−ΔΔCT)^ method described by [103].

**Table 2:** Sequence of genes and primers used for relative quantification by real-time PCR (qPCR) in hen’s uterine and liver tissues

Name of Target gene	Nucleotide sequence of primers (5′ → 3′)	Fragment Size (bp)	Reference (s)
**Oviduct genes**			
Ovocalyxin-32 (OCX32)	F: GGACAGCACTGCACTACATCAA	514	[38]
	R: GGAATTTCGTGGAGCAAGACAA		
Ovocalyxin-36 (OCX-36)	F: TTGGAATGGTCGTCTTCTGTGG	121	[39]
	R: CGGTCTGAATGATGGCATCG		
Ovocleidin-17 (OC-17)	F: CGTTCTGCCGCCGTTGGG	96	[40]
	R: CCCGCGACGCGTTGAGGA		
Ovocleidin-116 (OC-116)	F: AAGAGCCAACATCCAAGTGGGTGAGAAT	424	[41]
	R: CAGTGACCACATGGCTCCCTTTCCT		
**Hepatic selenoproteins**			
Glutathione peroxidase1	F: GCGACTTCCTGCAGCTCAACGA	99	[10, 11]
	R: CGTTCTCCTGGTGCCCGAAT		
Glutathione peroxidase4	F: CGGTGAATTACACTCAGCTCGT	123	
	R: CTTTGATCTGCGCGTCGTCC		
Iodothyronine deiodinase1	F: AAGCTGCACCTGACCTTCATT	138	
	R: TTGTTTCTGAAGGCCCATCCA		
Iodothyronine deiodinase4	F: CAGTGTAATCCACATAGCCA	137	
	R: CTGAGCCAAAATTAACCACC		
Selenoprotein W1	F: CTCCGCGTCACCGTGCTCT	155	
	R: CTGCCCACCGTCACCTCGAAC		
Thioredoxin reductase1.	F: ACTGGATGACTATGACCGAA	103	
	R: TATGCATTCTCATACGTGAC		
**Housekeeping**			
Glyceraldehyde-3-phosphate dehydrogenase	F: AATGAGAGGTTCAGGTGCCC	150	[10, 11]
	R: ACCAGACAGCACTGTGTTGG		
β-actin^8^	F: ACACACGGACACTTCAAGGG	128	
	R: TACTCAGCACCTGCATCTGC		
TATA-Box Binding Protein	F: TAGCCCGATGATGCCGTAT	147	[42, 43]
	R: GTTCCCTGTGTCGCTTGC		

### Statistical analysis

For reference gene validation, relative expression levels of all the target genes were calculated by the comparative 2^−ΔΔCq^ approach [103, 104], in Microsoft Excel (2016), using the two most stable reference genes (GAPDH and β-actin). From the excel, normalized relative quantities (NRQ) values were further analysed with One-way analysis of variance (ANOVA) using the Proc GLM procedure of SAS software (SAS Institute Inc., Cary, NC), and Duncan Multiple Range Test was used to separate level of significance (*p* < 0.05) between the treatment means. The results were presented as mean ± SEM.

## Data Availability

The datasets during and/or analyzed during the current study available from the corresponding author on reasonable request.

## Abbreviations

GPx: Glutathione peroxidase
TXNRD: Thioredoxin reductases
SELENOP: Selenoprotein P
SELENOW: Selenoprotein W
DIO: Iodothyronine deiodinase
Se: Selenium
Con: Control
Na_2_SeO_3_: Sodium selenite
Se-yeast: Selenium yeast
ADS18: *S. maltophilia*
RT-PCR: Real-time polymerase chain reaction
mRNA: Messenger RNA
qPCR: Quantitative real-time PCR
OC-17: Ovocleidin-17
OC-116: Ovocleidin-116
OCX-32: Ovocalyxin-32
OCX-36: Ovocalyxin-36
Ca^2+^: Calcium
HCO_3_^−^: Bicarbonate
BPI: Bactericidal permeability increasing
SSC: *S. cerevisiae*
CTS: Chitosan
Se-CTS-S: Selenide chitosan sulfate
CTS-S: Chitosan sulfate
CTS-Se: Selenide chitosan
SeMet: Selenomethionine
SeCys: Selenocysteine
HSP’s: Heat shock proteins
Ct: Cycle threshold
cDNA: Complementary DNA
NRQ: Normalized relative quantities
ANOVA: Analysis of variance
Q-Q: Quantile-QUANTILE
SEM: Standard error mean
